# High Resolution Melting Analysis for Rapid Mutation Screening in Gyrase and Topoisomerase IV Genes in Quinolone-Resistant *Salmonella enterica*

**DOI:** 10.1155/2014/718084

**Published:** 2014-10-12

**Authors:** Soo Tein Ngoi, Kwai Lin Thong

**Affiliations:** ^1^Institute of Biological Sciences, Faculty of Science, University of Malaya, 50603 Kuala Lumpur, Malaysia; ^2^Laboratory of Biomedical Science and Molecular Microbiology, Institute of Graduate Studies, University of Malaya, 50603 Kuala Lumpur, Malaysia

## Abstract

The increased* Salmonella* resistance to quinolones and fluoroquinolones is a public health concern in the Southeast Asian region. The objective of this study is to develop a high resolution melt curve (HRM) assay to rapidly screen for mutations in quinolone-resistant determining region (QRDR) of gyrase and topoisomerase IV genes. DNA sequencing was performed on 62* Salmonella* strains to identify mutations in the QRDR of* gyrA*,* gyrB*,* parC*, and* parE* genes. Mutations were detected in QRDR of* gyrA* (*n* = 52; S83F, S83Y, S83I, D87G, D87Y, and D87N) and* parE* (*n* = 1; M438I).* Salmonella* strains with mutations within QRDR of* gyrA* are generally more resistant to nalidixic acid (MIC 16 > 256 *μ*g/mL). Mutations were uncommon within the QRDR of* gyrB*,* parC*, and* parE* genes. In the HRM assay, mutants can be distinguished from the wild-type strains based on the transition of melt curves, which is more prominent when the profiles are displayed in difference plot. In conclusion, HRM analysis allows for rapid screening for mutations at the QRDRs of gyrase and topoisomerase IV genes in* Salmonella*. This assay markedly reduced the sequencing effort involved in mutational studies of quinolone-resistance genes.

## 1. Introduction

*Salmonella* is a human pathogen commonly found in developed and developing countries, causing clinical diseases ranging from mild gastroenteritis to septicaemia [1]. Quinolones are broad spectrum antimicrobial agents that inhibit bacterial DNA from unwinding and duplicating during cell division and are commonly used in treating* Salmonella* infection. However, the worldwide emergence of quinolone-resistant bacterial strains raises public health concern. Increased incidence of quinolone-resistant* Salmonella* serovars has been documented in Southeast Asian region [2–5].

Three major mechanisms contribute to quinolone-resistance among* Salmonella*: altered protein targets for quinolones, decreased uptake of quinolones by bacteria, and DNA gyrase protection* via* plasmid-derived* qnr* genes [6]. DNA gyrase and topoisomerase IV are two important enzymes involved in bacterial DNA replication. Quinolones bind to gyrase/topoisomerase IV-DNA complex and inhibit DNA replication. This action is responsible for the bacteriostatic and bactericidal property of quinolones. Mutations in the bacterial genes encoding DNA gyrase and topoisomerase IV may confer resistance to quinolones and it has been shown that altered structures of these enzymes prevent binding of quinolones [6, 7].

Both DNA gyrase and topoisomerase IV are tetrameric enzymes. DNA gyrase is encoded by* gyrA* and* gyrB* genes, while topoisomerase IV is encoded by* parC* and* parE* genes. Mutation in quinolone-resistant determining region (QRDR) in these genes is associated with quinolone-resistance [6]. Many studies have reported the presence of such mutations in quinolone-resistant* Salmonella* [8–12]. Indeed, lower fluoroquinolone susceptibility and quinolone-resistance are linked to point mutations in QRDR of the gyrase genes [13, 14]. While most studies revealed that codon 87 of* gyrA* gene represents the most frequently mutated amino acid in* Salmonella* [8, 12, 14], point mutations outside of QRDR may also play a role in quinolone-resistance [6]. Nevertheless, QRDR remains the major focus in quinolone-resistance studies since this region exhibits high mutation rate.

DNA sequencing is the gold standard for detecting genetic changes. However, it is relatively costly to sequence all four genes when examining a large sample pool. Furthermore, mutations are rare in* parC* and* parE* genes [9]; therefore DNA sequencing will not be an economically viable option when dealing with such conserved regions. The real-time, polymerase chain reaction- (PCR-) based high-resolution melt curve (HRM) analysis is able to detect small genetic variation in PCR amplicons [15–17]. This method is highly sensitive and specific and has been employed in single nucleotide polymorphism (SNP) scanning [18]. During HRM analysis, a DNA intercalating dye is included in the PCR reaction mixture. This compound interacts specifically with double-stranded DNA and emits fluorescence signal but loses fluorescence when released from the DNA during denaturation. Upon completion of PCR, the DNA samples are subject to a temperature gradient, and the loss of fluorescence signal resulting from denaturation gives each DNA sample a unique melting curve that is detected by the real-time PCR system [19]. Hence, different* Salmonella* strains can be discriminated based on their sequence, length, GC content, or strand composition.

HRM assay has been used for the identification of quinolone-resistance strains in pathogens such as* Mycobacterium tuberculosis*,* Bacillus anthracis*,* Yersinia pestis*,* Francisella tularensis*, and* Salmonella* Typhi and Paratyphi A [20–23]. In these studies, only* gyrA* gene was examined. Although infrequent, mutations in* gyrB*,* parC,* and* parE* genes of* Salmonella* and their correlation with lower susceptibility to quinolones and fluoroquinolones have been documented too [9]. The objective of the study was to determine the potential of HRM analysis for rapid screening of mutations in QRDR of* Salmonella* gyrase and topoisomerase IV genes.

## 2. Materials and Methods

### 2.1. Bacterial Strains

A total of 195* Salmonella enterica* strains were obtained from the culture collection of Laboratory of Biomedical Sciences and Molecular Microbiology, Institute of Graduate Studies, University of Malaya. The susceptibility of the strains towards nalidixic acid and ciprofloxacin was examined and reported in previous studies [24, 25]. Sixty-two* Salmonella* strains showing resistance or reduced susceptibility towards nalidixic acid were used to develop the HRM assay in this study (*Salmonella* Typhimurium, *n* = 12;* Salmonella* Enteritidis, *n* = 50). All selected strains were susceptible to ciprofloxacin. The minimum inhibitory concentration (MIC) of nalidixic acid for the strains was determined by using Etest strips (BioMérieux, Marcy l'Etoile, France). The test was performed according to manufacturer's instructions. Genomic DNA for each bacterial strain was extracted by using Wizard genomic DNA purification kit (Promega, Madison, USA). The details of all* Salmonella* strains used in this study are listed in the Supplementary Table (see Table S1 in the Supplementary Material available online at http://dx.doi.org/10.1155/2014/718084).

### 2.2. DNA Sequencing

PCR amplification of* gyrA*,* gyrB*,* parC*, and* parE* QRDRs was performed using previously described primers [26]. The selected primers amplify the QRDRs of the respective genes. PCR amplification was done in a 25 *μ*L monoplex reaction mixture, containing 1x colourless GoTaq Flexi Buffer, 1.5 mmol/L magnesium chloride (MgCl_2_), 200 *μ*mol/L deoxynucleoside triphosphate (dNTP) mix, 0.3 *μ*mol/L of each primer pair, 1 U* Taq* DNA polymerase (Promega, Madison, USA), and approximately 100 ng of bacterial genomic DNA. The PCR reaction mixtures were first incubated at 94°C for 2 min, followed by 25 cycles of 94°C for 30 s, 60–63°C (annealing temperature varies for each gene) for 30 s, and 72°C for 45 s, with a final extension step of 72°C for 5 min. The appropriate annealing temperatures for* gyrA*,* gyrB*,* parC*, and* parE* are 60°C, 60°C, 63°C, and 62°C, respectively. Agarose gel electrophoresis was then carried out to confirm the presence of amplicons prior to DNA sequencing. Next, the PCR products were purified using Wizard SV gel and PCR clean-up system (Promega, Madison, USA), and the purified PCR products were sent to First BASE Laboratories (Selangor, Malaysia) for sequencing. Mutations in the target genes were determined by comparison with the wild-type sequence of the respective genes in* Salmonella* Typhimurium LT2 (GenBank accession number NC_003197).

### 2.3. HRM Analysis

A homology search for* gyrA*,* gyrB*,* parC*, and* parE* gene sequences of* Salmonella* serovars from the NCBI GenBank database was performed. Sequence alignment was done using the molecular evolutionary genetics analysis software version 5 (MEGA5) [27]. Primers were designed using the online tool Primer3Plus (http://primer3plus.com/cgi-bin/dev/primer3plus.cgi), spanning the QRDR of each gene of interest, and were commercially synthesized (NHK Bioscience Solutions, Kuala Lumpur, Malaysia). Approximately 20 ng of bacterial genomic DNA was added into 1x MeltDoctor HRM master mix (Applied Biosystems, Foster City, CA, USA), which was premixed with 0.3 *μ*M of each primer pair. Sterile deionized distilled water was added to make up a final reaction volume of 20 *μ*L. The reproducibility of the assay was determined by performing all reactions in triplicate. In each assay, the DNA sample from a wild-type reference strain was used as positive control. The selection of the wild-type reference strains for the different target genes was explained in the Results section (Section 3.2). Negative control for the experiment was the HRM reaction mix without the addition of bacterial DNA. The HRM assay was performed on the 7500 Fast Real-Time PCR System (Applied Biosystems). The real-time PCR amplification included a holding stage at 95°C for 10 min, followed by the cycling stage with 40 cycles of 95°C for 15 sec and subsequently 60°C for 1 min. The subsequent melt curve stage consisted of the following steps: 95°C for 15 sec, a melt from 60°C (1 min) to 95°C (30 sec) with a ramp rate of 1%, and 60°C for 15 sec (according to manufacturer's instructions). The amplification plot for each sample was generated by the 7500 software v2.0 (Applied Biosystems) linked to the instrument. The data generated was subsequently imported to the HRM software v2.0.1 (Applied Biosystems) for melting curve analysis. The fluorescence change of each sample was plotted against temperature and was normalized to produce the aligned melt curves. In order to better illustrate the minor fluorescence changes between the wild type and mutants, a difference plot was generated using the HRM software. In a difference plot, one* Salmonella* strain with wild-type sequence confirmed via sequencing was chosen as a reference. The fluorescence difference between each sample and the reference was plotted against temperature. Mutations in the target region would produce a deviated curve in the difference plot.

## 3. Results 

### 3.1. Detection of QRDR Mutations by Sequencing

The* gyrA*,* gyrB*,* parC*, and* parE* genes of 62* Salmonella* strains were subjected to sequencing. Of these 62 strains, missense mutations were detected in QRDR of* gyrA* (*n* = 52) and* parE* (*n* = 1). Silent mutations within and beyond QRDR were observed in* gyrB* (*n* = 60),* parC* (*n* = 1), and* parE* (*n* = 50). In QRDR of* gyrA*, missense mutations occurred only in codons 83 (conversion of serine to phenylalanine (S83F), tyrosine (S83Y), and isoleucine (S83I)) and 87 (conversion of aspartic acid to glycine (D87G), tyrosine (D87Y), and asparagine (D87N)). In the* parE* gene, two missense mutations were detected, one within the QRDR (methionine to isoleucine (M438I)) and another outside of the QRDR (valine to phenylalanine (V521F)) (Table 1). The MIC data for all 62 strains are shown in Table 1. In general, mutations at the QRDR of* gyrA* correlate with increased resistance to nalidixic acid (MIC 16–>256 *μ*g/mL). In the absence of mutations (STM057/05), the strain showed lowest MIC value (MIC 6 *μ*g/mL). Detailed genetic analyses of the QRDRs of each* Salmonella* strain are listed in the Supplementary Table (Table S1).

### 3.2. HRM Analysis for Rapid Mutation Screening of Gyrase and Topoisomerase IV Genes

HRM primers were designed to span the QRDRs of* gyrA* (Ala67-Gln106),* gyrB* (Asp426-Lys447),* parC* (Ala64-Gln103), and* parE* (Asp420-Lys441) genes. The primer pairs produce amplicons ranging from 150 to 250 bp (Table 2), with a melting temperature of approximately 60°C. HRM analysis showed distinct melt curves for wild-type versus mutant strains, which were confirmed by sequencing. Using sequencing as a guideline, two QRDR mutation-free strains were selected as references to generate difference plots (STM006/02 for* gyrA*,* parC*, and* parE* genes; STM032/04 for* gyrB* gene). The QRDRs of* gyrA*,* parC*, and* parE* genes in the reference strain STM006/02 and the* gyrB* QRDR in the reference strain STM032/04 have matching sequences with the* Salmonella* Typhimurium reference genome LT2 (NC_003197). Detailed information for these two reference strains is listed in the Supplementary Table (Table S1).

**Figure 1:**
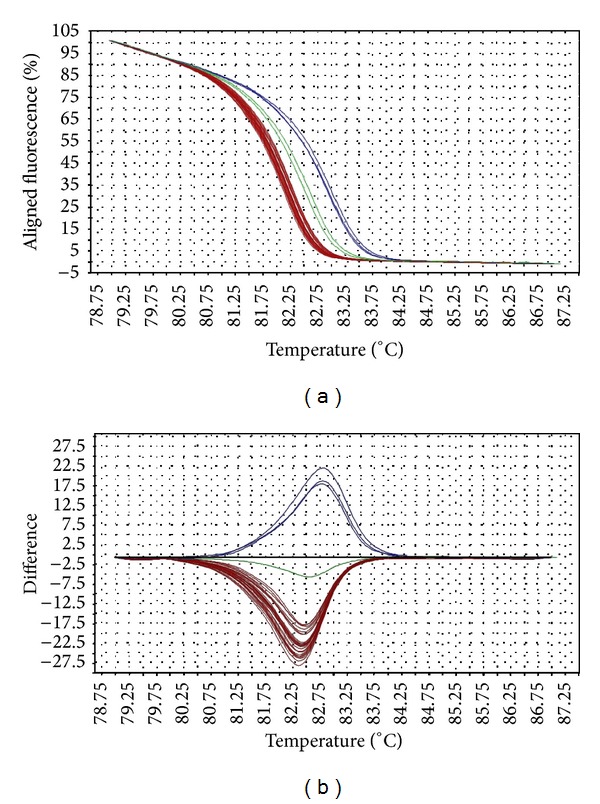
Representative HRM aligned melting curves (a) and difference plot (b) for mutations in* gyrA* QRDR. The aligned melting curves were set at 100% at the beginning and 0% at the end of melting process. In the difference plot, the melting curves represent the temperature at which the amplicons were completely denatured. The difference of the melting temperature among the samples is clearly illustrated in the difference plot. The reference strain is indicated by the horizontal black line in the difference plot; and the wild-type samples are indicated as green line. The blue curves derived from mutants with the missense mutation D87G, whereas the red curves belong to other mutants (D87Y, D87N, S83F, and S83Y). Mutants with missense mutations D87Y, D87N, S83F, and S83Y were denatured at similar melting temperature and therefore form a tight cluster.

**Figure 2:**
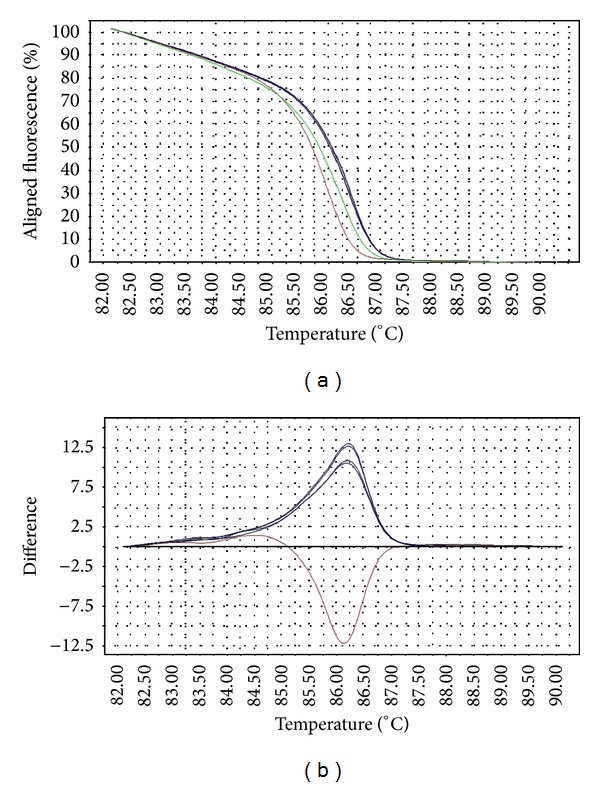
Representative HRM aligned melting curves (a) and difference plot (b) for mutations in* gyrB* QRDR. The reference strain is indicated by the horizontal black line in the difference plot, while mutants are represented by red (6 mutations within the HRM target region) or blue curves (3 mutations within the HRM target region). All* gyrB* mutants are silent mutations.

**Figure 3:**
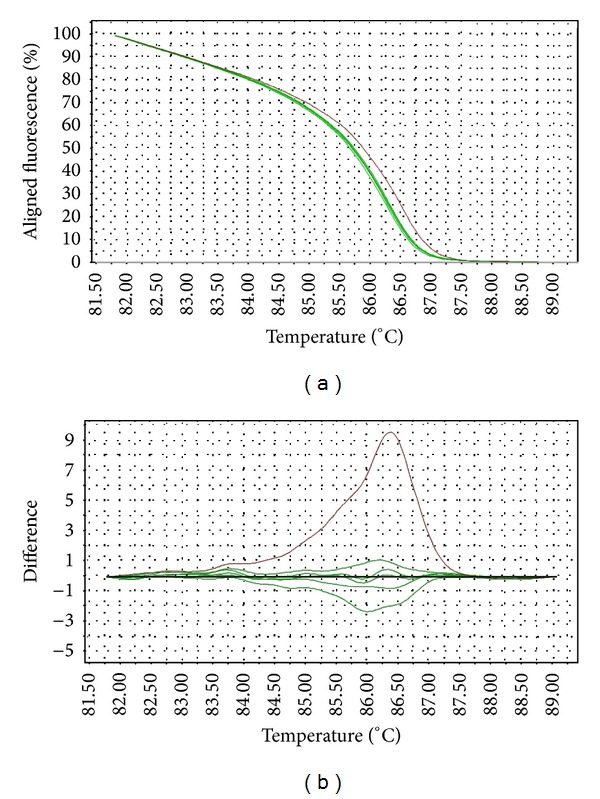
Representative HRM aligned melting curves (a) and difference plot (b) for mutations in* parC* QRDR. The reference strain is indicated by the horizontal black line in difference plot. Wild-type samples are indicated by green curves and the single mutant is indicated by red curve.

**Figure 4:**
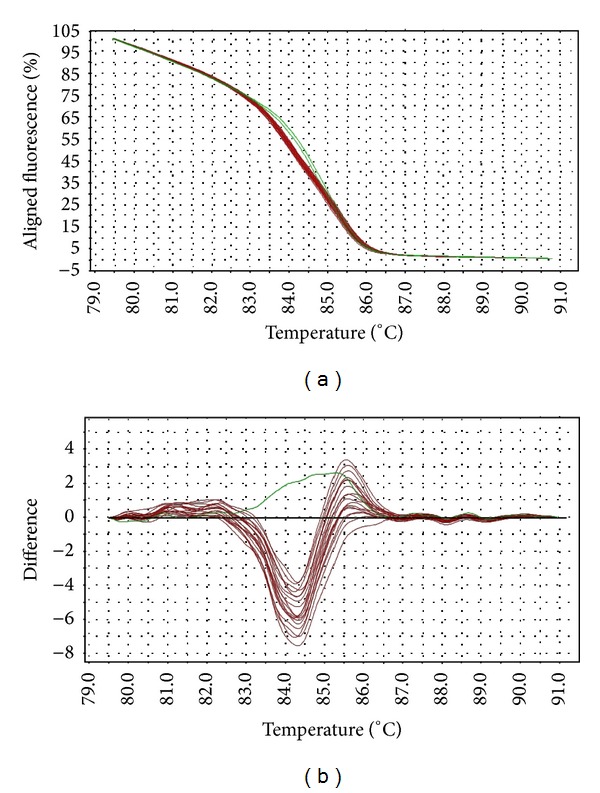
Representative HRM aligned melting curves (a) and difference plot (b) for mutations in* parE* QRDR. The reference strain is indicated by the horizontal black line in difference plot. Wild-type samples are indicated by green curves and mutants are indicated by red curves. All mutant strains contain similar nucleotide changes in the QRDR, resulting in tightly clustered melting curves.

The melting temperature of wild-type* gyrA* allele is 82.5–82.8°C, while that of the mutants (D87Y, D87N, S83F, and S83Y) is at 82.0–82.4°C. Interestingly,* Salmonella* strains with* gyrA* D87G mutation produced a distinct melting curve compared to other mutants, at slightly higher melting temperature (82.9–83.5°C). The differences in the melting curves of the wild type versus mutants were distinguishable in both aligned and difference plot (Figure 1). The melting temperature for* gyrB* wild-type allele is 86.1°C–86.2°C, while that of the mutants is 86.2–86.7°C. One mutant (STM043/05) with three mutations in* gyrB* has a slightly lower melting temperature at 86.0°C. The aligned plot did not show any difference when comparing the melt curves of wild-type versus mutant* gyrB* alleles. However, they can be distinguished in difference plot (Figure 2). For* parC* gene, the melting temperature for wild-type allele is at 86.1–86.5°C (98% melted below 86.4°C) and the mutant at 86.4°C. Although the differences in the melting temperature were small, the mutant produced a unique melting curve in the difference plot when compared to the wild type (Figure 3). For* parE* gene, the melting temperature for wild-type allele is at 84.6–84.9°C, whereas that of the mutants containing multiple mutations is at 84.9–85.2°C. One mutant (STM018/03), which harbors a single mutation in* parE* QRDR, has a lower melting temperature at 83.9°C. The differences in wild-type versus mutant melt curves were apparent in both aligned and difference plot (Figure 4).

## 4. Discussion

The HRM primers designed in this study spanned the entire QRDR of all four target genes. The QRDRs are short regions of DNA, making them suitable targets for HRM analysis (optimal amplicon length 150–350 bp). The transition of melt curves in HRM analysis of all target regions successfully discriminated the wild types from mutants. Although the melting transitions were not very distinct in aligned melt curves, especially for* gyrB*, the melting transitions of the mutants became apparent when displayed in a difference plot. These transitions are direct consequences of the mutations in the target gene QRDRs, since they are the only genetic variation within the PCR region.

Point mutations were identified in QRDRs of* gyrA*,* gyrB*,* parC,* and* parE*. Consistent with the literature [9, 14], our study identified that mutations in* gyrA* QRDR most commonly take place at codons 83 and 87, often resulting in amino acid change, and are associated with the nalidixic acid resistance in* Salmonella* strains. Gyrase A-DNA complex is the primary target for quinolones in* Salmonella* [7]. Hence, prolonged usage of quinolones often selects for development of resistant strains with mutations in* gyrA*, since such mutations confer better resistance to quinolone compared to that of* gyrB* mutants [9]. HRM analysis of the* gyrA* successfully resolved the mutant strains into three distinct melting profiles. The melting profiles of the D87G mutants were highly divergent from those of the other mutants. The D87G variants consisted of a single base substitution from adenosine to guanine. This type of mutation represents class I SNP, which often results in a higher melting temperature shift compared to other classes of SNP.

Mutation was not common in the QRDR of* gyrB* among* Salmonella* strains in this study. Furthermore, most studies did not identify mutations in* gyrB* of quinolone-resistant* Salmonella* [9, 14, 28]. The HRM primers designed in this study covered a region larger than the QRDR, with an additional 132 bp. Hence, the HRM variants detected in this study consisted of three point mutations outside of QRDR regions. However, all nucleotide changes resulted in silent mutations. One strain (STM043/05) consisted of a silent mutation in the QRDR and an additional three mutations compared to other mutants. This strain consisted of class I SNPs at two sites, thus producing a melting profile different from other variants. The different variants can be resolved by HRM as distinct difference plots.

Topoisomerase is the secondary target of quinolones in Gram-negative bacteria. Mutations within QRDRs of* parC* and* parE* genes are uncommon among* Salmonella* [9, 14, 28]. In this study, amino acid changes were not detected in* parC* genes and were identified in* parE* gene of only two* Salmonella* strains. The strains used in this study showed classical phenotype, that is, resistant to nalidixic acid but not to ciprofloxacin. These observations are in agreement with previous reports, which suggested that the mutations of these genes are most probably infrequently detected because they either are not important in quinolone-resistance or are only required for higher level of resistance, that is, resistance to fluoroquinolones [9]. Multiple mutations were detected both within and beyond the QRDRs of* parC* and* parE* genes. However, these were mostly silent mutations. HRM assay successfully differentiated the mutants from the wild type. The melting profiles of the mutants were highly divergent from those of the wild type, as clearly shown in both aligned melt curves and difference plots.

Despite the high efficiency of HRM analysis in mutation screening, this assay could only detect the presence of base substitutions. The melting profiles should not be used directly for variants classification without first validating with DNA sequencing. False positive may occur due to synonymous mutations, while false negative occurs due to mutations outside targeted regions or resistance caused by other mechanisms. To date, sequencing remains the ultimate proof for mutations that lead to amino acid changes and subsequently an alteration of enzyme structure [29]. Nevertheless, melting profiles are useful parameters to distinguish mutants with different characteristic and number of mutations, since individual mutant presents a distinctive melt curve when viewed in the difference plot. Hence, HRM analysis can be considered as an efficient and cost-effective preliminary step in the process of identifying QRDR mutations in* Salmonella*.

In conclusion, HRM analysis allows for detection of mutations in* Salmonella* gyrase and topoisomerase IV genes with sufficient sensitivity. The adoption of this assay for mutation screening prior to DNA sequencing is an attractive option to reduce the cost for mutational studies related to quinolone resistance. Furthermore, HRM analysis is time-saving, allows for high throughput screening, and is easy to perform since there is no post-PCR processing or purification steps. Nevertheless, sequencing shall be used for final validation and should not be completely replaced by HRM analysis.

## Supplementary Material

The background of the *Salmonella* strains used to develop the HRM assay is tabulated in the supplementary table (Table S1). Additional information include the susceptibility of each *Salmonella* strain to nalidixic acid and the corresponding minimum inhibitory concentration value, type of base substitution and the corresponding amino acid change in each strain, and the melting temperature of the HRM amplicons.

## Figures and Tables

**Table 1:** Mutations in the gyrase and topoisomerase IV genes as determined by DNA sequencing and the nalidixic acid minimum inhibitory concentration (MIC) of their corresponding *Salmonella* strains.

Strain	MIC (*μ*g/mL)	*gy* *rA* ^a^	*gyrB *	*parC *	*pa* *rE* ^a^
SE 109/07	>256	D87Y	Silent mutations	None	V521F
SE 005/03	>256	D87Y	Silent mutations	None	Silent mutations
SE 022/03	>256	D87Y	Silent mutations	None	Silent mutations
SE 026/03	>256	D87Y	Silent mutations	None	Silent mutations
SE 027/03	>256	D87Y	Silent mutations	None	Silent mutations
SE 028/03	>256	D87Y	Silent mutations	None	Silent mutations
SE 032/03	>256	D87Y	Silent mutations	None	Silent mutations
SE 033/03	>256	D87Y	Silent mutations	None	Silent mutations
SE 047/04	>256	D87Y	Silent mutations	None	Silent mutations
SE 054/05	>256	D87Y	Silent mutations	None	Silent mutations
SE 064/05	>256	D87Y	Silent mutations	None	Silent mutations
SE 066/05	>256	D87Y	Silent mutations	None	Silent mutations
SE 067/05	>256	D87Y	Silent mutations	None	Silent mutations
SE 069/05	>256	D87Y	Silent mutations	None	Silent mutations
SE 073/05	>256	D87Y	Silent mutations	None	Silent mutations
SE 075/05	>256	D87Y	Silent mutations	None	Silent mutations
SE 078/05	>256	D87Y	Silent mutations	None	Silent mutations
SE 080/05	>256	D87Y	Silent mutations	None	Silent mutations
SE 081/05	>256	D87Y	Silent mutations	None	Silent mutations
SE 086/06	>256	D87Y	Silent mutations	None	Silent mutations
SE 101/07	>256	D87Y	Silent mutations	None	Silent mutations
SE 104/07	>256	D87Y	Silent mutations	None	Silent mutations
SE 105/07	>256	D87Y	Silent mutations	None	Silent mutations
SE 106/07	>256	D87Y	Silent mutations	None	Silent mutations
SE 107/07	>256	D87Y	Silent mutations	None	Silent mutations
SE 002/03	192	D87Y	Silent mutations	None	Silent mutations
SE 076/05	192	D87Y	Silent mutations	None	Silent mutations
SE 004/03	128	D87Y	Silent mutations	None	Silent mutations
SE 048/04	128	D87Y	Silent mutations	None	Silent mutations
SE 097/06	128	D87Y	Silent mutations	None	Silent mutations
SE 074/05	96	D87Y	Silent mutations	None	Silent mutations
SE 090/06	96	D87Y	Silent mutations	None	Silent mutations
SE 068/05	64	D87Y	Silent mutations	None	Silent mutations
SE 102/07	64	D87Y	Silent mutations	None	Silent mutations
SE 046/04	48	D87Y	Silent mutations	None	Silent mutations
SE 049/04	48	D87Y	Silent mutations	None	Silent mutations
SE 084/05	24	D87Y	Silent mutations	None	Silent mutations
SE 001/03	16	D87Y	Silent mutations	None	Silent mutations
SE 065/05	16	D87Y	Silent mutations	None	Silent mutations
STM 032/04	>256	D87G	None	None	None
SE 089/06	>256	D87G	Silent mutations	None	Silent mutations
SE 095/06	>256	D87G	Silent mutations	None	Silent mutations
SE 070/05	128	D87G	Silent mutations	None	Silent mutations
SE 050/04	64	D87G	Silent mutations	None	Silent mutations
SE 012/03	>256	D87N	Silent mutations	None	Silent mutations
SE 015/03	>256	D87N	Silent mutations	None	Silent mutations
SE 013/03	96	D87N	Silent mutations	None	Silent mutations
STM 043/05	>256	S83F	Silent mutations	Silent mutations	Silent mutations
SE 055/05	>256	S83F	Silent mutations	None	Silent mutations
SE 051/04	>256	S83I	Silent mutations	None	Silent mutations
SE 052/04	>256	S83I	Silent mutations	None	Silent mutations
SE 099/06	>256	S83Y	Silent mutations	None	Silent mutations
STM 018/03	24	None	Silent mutations	None	M438I
STM 071/07	64	None	Silent mutations	None	None
STM 055/05	32	None	Silent mutations	None	None
STM 006/02	16	None	Silent mutations	None	None
STM 033/04	16	None	Silent mutations	None	None
STM 053/05	16	None	Silent mutations	None	None
STM 056/05	16	None	Silent mutations	None	None
STM 034/04	12	None	Silent mutations	None	None
STM 048/05	8	None	Silent mutations	None	None
STM 057/05	6	None	None	None	None

D, aspartic acid; F, phenylalanine; G, glycine; I, isoleucine; M, methionine; N, asparagine; S, serine; V, valine; Y, tyrosine; “silent mutations” denotes synonymous base substitutions in the QRDR of the target genes and thus does not result in amino acid change; “none” denotes wild-type sequence in the QRDR; that is, base substitution did not take place.

^a^The first letter indicates the original amino acid, followed by numbers that indicate the position of the amino acid in respective gene, and the last letter indicates the substituting amino acid. For example, D87Y means tyrosine replaced aspartic acid in position 87 in GyrA subunit.

**Table 2:** PCR primer sequences for HRM analysis of gyrase and topoisomerase IV genes.

Primer	Sequence (5′-3′)^b^	Amplicon size (bp)
gyrA-F	CAATGACTGGAACAAAGCCTA	164
gyrA-R	AACCGAAGTTACCCTGACCA	
gyrB-F	TGTCCGAACTGTACCTGGTG	198
gyrB-R	ACTCGTCGCGACCGATAC	
parC-F	CGTCTATGCGATGTCAGAGC	219
parC-R	ATCGCCGCGAATGACTTC	
parE-F	TACCGCGCAGGATCTTAATC	193
parE-R	GATCGCCACGGAAATATCAT	

^b^Primers designed in this study.
